# Correlation Between Sex Hormones (Testosterone and Prolactin) and Anti-ds-DNA Antibody Levels in Systemic Lupus Erythematosus Patients: A Gender-Specific Analysis of the Hormonal Influence on Immune Responses

**DOI:** 10.7759/cureus.70049

**Published:** 2024-09-23

**Authors:** Khalid Elgorashi, Mahda Omer, Enas Alhaj, Samia Omer Ali

**Affiliations:** 1 Internal Medicine, University of Khartoum, Khartoum, SDN; 2 Internal Medicine, Ahfad University for Women, Omdurman, SDN; 3 Immunology, Al-Rayan Specialized Medical Laboratory Complex, Sudan Academy of Sciences, Khartoum, SDN; 4 Immunology, Sudan Academy of Sciences, Khartoum, SDN

**Keywords:** anti-ds-dna antibodies, gender differences, prolactin, systemic lupus erythematosus, testosterone

## Abstract

Background

Systemic lupus erythematosus (SLE) is a chronic autoimmune disease that mostly affects women and has a noteworthy gender gap. Although the precise effects of sex hormones on SLE are yet unknown, they may modulate the immune response and affect the severity of the illness. Two important sex hormones that have a possible influence on autoimmune processes are prolactin and testosterone. The objective of this study was to assess the correlation between the levels of different sex hormones (testosterone and prolactin) and anti-double-stranded DNA (anti-ds-DNA) antibody levels in Sudanese anti-ds-DNA-positive SLE patients.

Methodology

A prospective cross-sectional study was conducted from February to July 2017 at Al-Rayan Specialized Medical Laboratory Complex and Omdurman Military Hospital, Sudan. There were 90 SLE patients who tested positive for anti-ds-DNA. Using commercial kits, serum levels of prolactin, testosterone, and anti-ds-DNA antibodies were tested from blood samples. SPSS version 21 was used for statistical analysis to find relationships between hormone levels and antibody levels.

Results

In this study of 90 patients with SLE, gender distribution was 14 males (16%) and 76 females (84%). The mean prolactin levels were significantly higher in males (17.99 ng/ml) compared to females (15.73 ng/ml, P < 0.001), and testosterone levels were also significantly higher in males (1.43 ng/ml) than females (0.37 ng/ml, P = 0.001). Although males had slightly higher mean levels of anti-ds-DNA antibodies (4.51) compared to females (4.08), this difference was not statistically significant. The results of correlation analyses indicated a weak and non-significant link between anti-ds-DNA antibody levels and hormone levels, with prolactin's Spearman's rho value of -0.054 (P = 0.616) and testosterone's Spearman's rho value of 0.080 (P = 0.456).

Conclusion

It was found that prolactin and testosterone levels differ by gender in SLE patients and that these hormones may have an impact on how the condition is managed. But the poor associations with anti-ds-DNA antibody levels suggest that further investigation is required to fully comprehend these intricate interactions.

## Introduction

The complicated autoimmune disease known as systemic lupus erythematosus (SLE) is characterized by systemic inflammation and dysfunction of the immune system [1,2]. A complex interaction between genetic, environmental, and hormonal variables plays a role in its etiology [3]. Although SLE may impact people from a wide range of demographic backgrounds, women are disproportionately affected in terms of prevalence [4,5]. This gender difference raises the possibility that sex hormones are crucial in controlling immunological responses in individuals with SLE [6].

Two important sex hormones that have drawn interest for their possible influence on autoimmune processes are prolactin and testosterone [7]. In addition to its well-known effects on male reproduction, testosterone has also been linked to immune response modulation, which may have an impact on the severity and course of autoimmune disorders [8]. On the other hand, prolactin, a hormone with the well-established involvement in breastfeeding and reproductive processes, has been connected to inflammatory responses and immune system regulation [9]. Although these hormones have been shown to be significant in a number of autoimmune diseases, their precise involvement in SLE is still unknown [10,11].

Particularly in a gender-specific setting, the connection between sex hormones and anti-double-stranded DNA (anti-ds-DNA) antibodies, a defining feature of SLE pathogenesis, has not been well studied [12]. Anti-ds-DNA antibodies are essential to the diagnosis of the illness and are often linked to organ damage and disease activity [13]. Gaining knowledge about the correlation between these antibodies and levels of prolactin and testosterone may help identify mechanisms unique to each gender that either protect against or exacerbate the illness [14].

Even while the literature on sex hormones and autoimmune disorders is expanding, little is known about how they affect anti-ds-DNA antibody levels in SLE patients, especially in patients from certain groups like Sudanese people. Closing this gap might help understand how differences in hormones affect the variability and development of illness in different genders.

Research objective

The objective of study was to assess the correlation between the levels of different sex hormones (testosterone and prolactin) and anti-ds-DNA antibody levels in Sudanese anti-ds-DNA-positive SLE patients.

## Materials and methods

Study design and settings

This research examined the relationship between anti-ds-DNA antibody levels and sex hormones (prolactin and testosterone) in individuals with SLE using a prospective cross-sectional descriptive methodology. The study was carried out at Omdurman Military Hospital and Al-Rayan Specialized Medical Laboratory Complex during February to July 2017. Based on their prior SLE diagnosis, which was verified by clinical records in accordance with the updated American College of Rheumatology criteria, participants were chosen from these institutions.

Inclusion and exclusion criteria

Patients having a verified positive anti-ds-DNA antibody status, a confirmed diagnosis of SLE, satisfying at least four of the new American College of Rheumatology criteria, 18 years of age or older, and giving informed verbal consent were included in the research study. Patients with unfinished or missing medical data, those with coexisting diseases that might alter antibody or hormone levels, pregnant women, and those with hormonal abnormalities unrelated to SLE were excluded.

Sample size

The sample size was based on the number of eligible patients identified during the study period. A total of 90 patients were enrolled, meeting the study's inclusion criteria.

Data collection

Blood samples (3 ml) were collected from each participant via venipuncture, into plain tubes. After allowing the blood to clot, the samples were centrifuged to obtain serum, which was then stored at -20°C until analysis. Quantitative and qualitative analyses of the serum were performed using EUROIMMUN IgG anti-ds-DNA kits (Lübeck, Germany) and hormone kits from Fortress Diagnostics (Antrim, United Kingdom). The testing procedure involved warming the kits and samples to room temperature, mixing, and diluting samples in a 1:20 ratio in a sample buffer. Serum levels of anti-ds-DNA antibodies, testosterone, and prolactin were measured following the manufacturers’ instructions. Enzyme conjugate and chromogen substrate solutions were used, and the optical density was measured at 450 nm with reference wavelengths between 620 and 650 nm.

Statistical analysis

IBM SPSS, version 21 (IBM Corp., Armonk, NY) was used to analyze the data. Appropriate statistical methods were used to evaluate correlations between anti-ds-DNA antibody levels and sex hormone levels. With a 95% confidence interval, a P-value of less than 0.05 was considered statistically significant.

Ethical approval

The research was approved by the Khartoum State Ministry of Health's Ethical Review Committee, in accordance with ethical guidelines. Before being included in the study, participants gave verbal informed permission; ethical standards were followed throughout the investigation.

## Results

The study included a total of 90 patients, with a gender distribution of 14 males (16%) and 76 females (84%). This indicates a higher prevalence of female patients compared to males in the sample. The age distribution of the study's male and female patients is shown in Table 1, with patients divided into the following age groups: 3 males (21.4%) and 10 females (13.2%) under 20 years old; 8 males (57.1%) and 49 females (64.5%) between 20 and 40 years old, and 3 males (21.4%) and 17 females (22.4%) over 41 years old. This table gives an overview of the gender distribution in relation to the various age categories in the study population and emphasizes the preponderance of females in all age groups, especially in the 20- to 40-year-old range.

**Table 1:** Age distribution of male and female patients with systemic lupus erythematosus

Age group (years)	Male, n (%)	Female, n (%)
<20	3 (21.4%)	10 (13.2%)
20-40	8 (57.1%)	49 (64.5%)
≥41	3 (21.4%)	17 (22.4%)
Total	14 (100%)	76 (100%)

**Figure 1:**
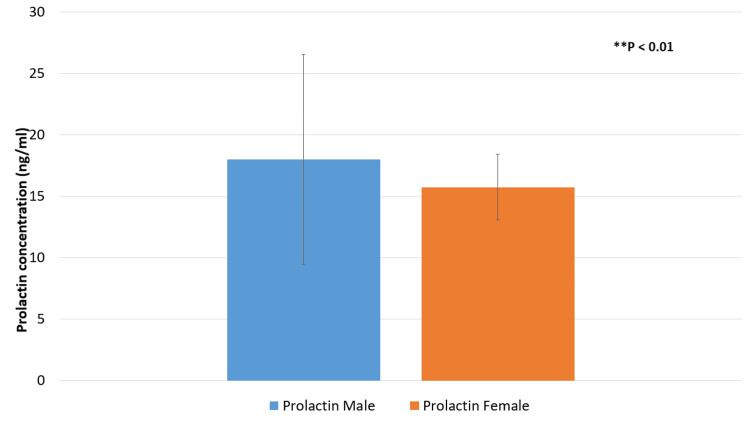
Mean prolactin concentrations according to gender ^**^P < 0.01 is significant.

Mean prolactin concentrations for male and female patients are shown in Figure 1. The mean prolactin level was 17.99 ng/ml in males and in females was 15.73 ng/ml. This difference was statistically significant (P < 0.001).

**Figure 2:**
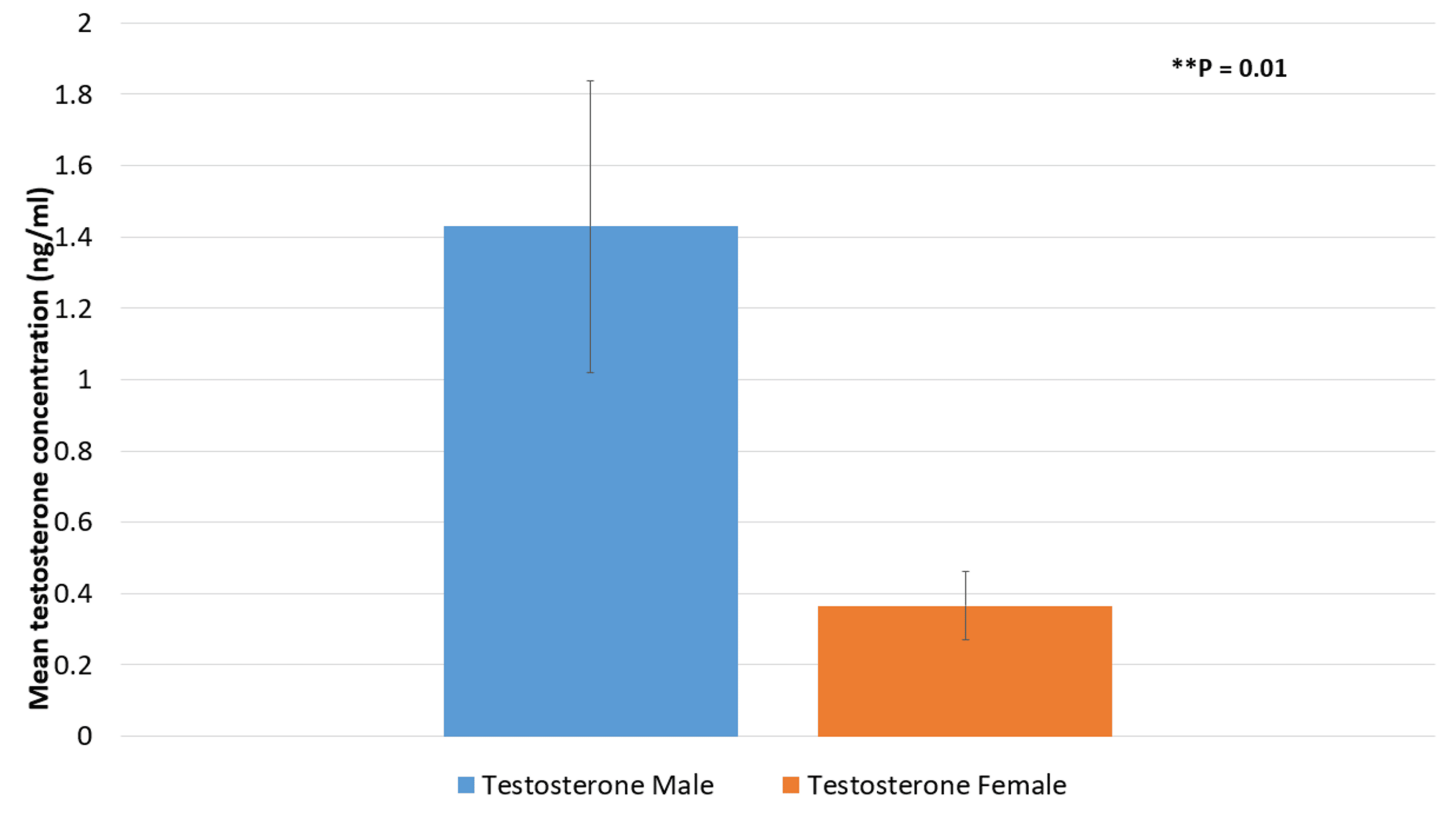
Mean testosterone concentrations according to gender ^**^P < 0.01 is significant.

Mean testosterone concentrations for male and female patients are shown in Figure 2. A P-value of 0.001 indicates that the mean testosterone level in males was substantially greater (1.4286 ng/ml) than in females (0.3658 ng/ml), indicating the differences in hormones between genders.

**Figure 3:**
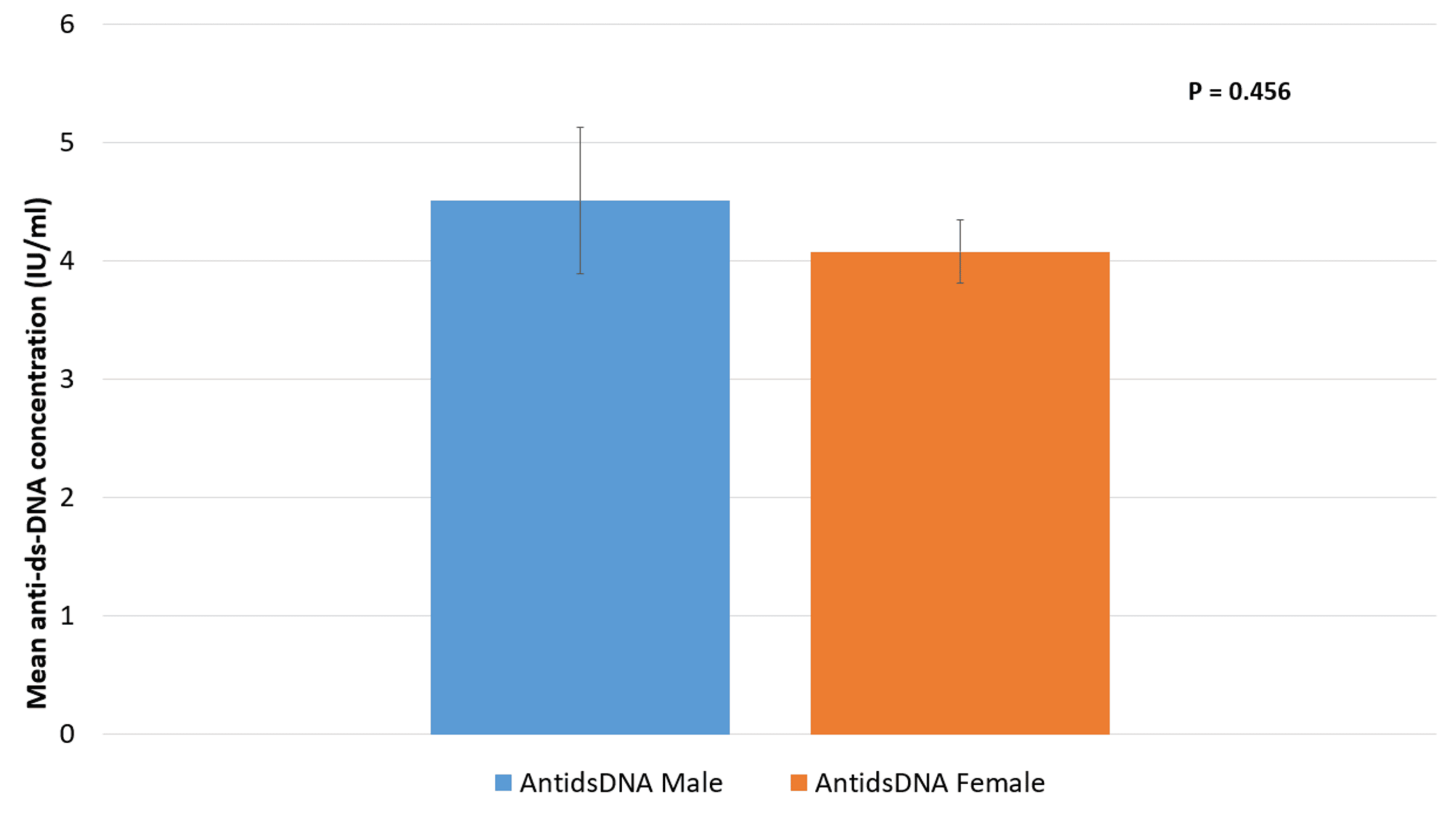
Mean anti-ds-DNA levels by ratio according to gender P > 0.05 is non-significant.

The mean levels of anti-ds-DNA antibodies in males and females are shown in Figure 3. The mean level in males was somewhat higher (4.507) than that in females (4.076), but this difference was not statistically significant.

Means, standard deviations, and confidence intervals for levels of prolactin, testosterone, and anti-ds-DNA in study participants are shown in Table 2. Only the differences in prolactin and testosterone levels were statistically significant, despite the fact that men had higher mean levels of prolactin (17.993 ng/ml), testosterone (1.4286 ng/ml), and anti-ds-DNA (4.507) than women (15.730 ng/ml, 0.3658 ng/ml, and 4.076, respectively).

**Table 2:** Prolactin, testosterone, and anti-ds-DNA levels (ratio) in study participants

Variables		N	Mean	Std. deviation	Std. error	95% confidence interval		Minimum	Maximum
						Lower bound	Upper bound		
Prolactin	Male	14	17.993	31.9031	8.5265	-0.427	36.413	1.9	124.5
	Female	76	15.730	23.3152	2.6744	10.403	21.058	1.1	131.6
	Total	90	16.082	24.6462	2.5979	10.920	21.244	1.1	131.6
Testosterone	Male	14	1.4286	1.52489	0.40754	0.5481	2.3090	0.10	5.00
	Female	76	0.3658	0.83067	0.09528	0.1760	0.5556	0.10	7.10
	Total	90	0.5311	1.03497	0.10910	0.3143	0.7479	0.10	7.10
Anti-ds-DNA	Male	14	4.507	2.3239	0.6211	3.165	5.849	1.2	8.5
	Female	76	4.076	2.3274	0.2670	3.544	4.608	1.2	10.0
	Total	90	4.143	2.3191	0.2445	3.658	4.629	1.2	10.0

The Spearman correlation analysis in the table highlights the relationships between anti-ds-DNA antibody levels and serum prolactin and testosterone levels in male and female subjects. Among male participants, a very weak negative correlation was found between anti-ds-DNA and prolactin levels (r = -0.063, P = 0.825), indicating no significant association. Additionally, the correlation between anti-ds-DNA and testosterone levels in males was also weak and not statistically significant (r = 0.102, P = 0.736) (Table 3). These findings suggest that neither prolactin nor testosterone levels are meaningfully related to anti-ds-DNA antibody levels in male patients with SLE, as evidenced by the low correlation coefficients and high P-values.

**Table 3:** Correlation between anti-ds-DNA antibody levels and serum prolactin and testosterone levels by gender P < 0.05 was significant.

Parameter		Anti-ds-DNA (male), N=1.0004	Prolactin (male), N=1.0004	Testosterone (male), N=1.0004	Anti-ds-DNA (female), N=76	Prolactin (female), N=76	Testosterone (female), N=76
Anti-ds-DNA (male)		1.000	-0.063	0.1.00002	-	-	-
Prolactin (male)		-0.063	1.000	-	-	-	-
Testosterone (male)		0.1.00002	-	1.000	-	-	-
P-value (male)	-		0.825	0.736	-	-	-
Anti-ds-DNA (female)		-	-	-	1.000	-0.047	0.08
Prolactin (female)		-	-	-	-0.047	1.000	-
Testosterone (female)		-	-	-	0.08	-	1.000
P-value (female)		-	-	-	0.697	-	0.456

Similarly, in female participants, results showed weak, non-significant correlations between anti-ds-DNA and both prolactin (r = -0.047, P = 0.697) and testosterone (r = 0.080, P = 0.456) (Table 3). This indicates no meaningful relationship between these hormone levels and anti-ds-DNA antibodies in females. Overall, these findings suggest that there is no significant correlation between anti-ds-DNA antibody levels and hormone concentrations (prolactin and testosterone) in either gender. Therefore, the results do not support the hypothesis that hormonal fluctuations in prolactin or testosterone have a significant impact on the levels of anti-ds-DNA antibodies in SLE patients.

## Discussion

This study's findings demonstrate that there are notable gender-specific variations in the relationship between sex hormones and anti-ds-DNA antibody levels in SLE patients. The research sample showed a significant gender imbalance, with 76 females (84%) and 14 men (16%). This distribution of demographic characteristics is consistent with earlier research results that indicate women have SLE at a higher rate than males, perhaps as a result of sex hormones having an impact on the pathophysiology of the illness [15,16]. Our research demonstrated significant gender differences in mean prolactin and testosterone concentrations. Specifically, men had a mean testosterone level of 1.4286 ng/ml compared to 0.3658 ng/ml in females (P = 0.001), and a mean prolactin level of 17.99 ng/ml compared to 15.73 ng/ml in females (P < 0.001). These findings align with previous studies that highlight considerable gender-based variations in hormone levels and their potential impact on autoimmune disease activity [17,18]. The higher testosterone levels and elevated prolactin in males suggest that hormonal differences may influence immunological responses in SLE, potentially modulating disease activity based on gender.

In comparison to females, who had mean levels of 15.730 ng/ml and 0.3658 ng/ml, respectively, men had higher mean levels of prolactin (17.993 ng/ml) and testosterone (1.4286 ng/ml) according to our research. Prior research has emphasized the function of testosterone in immune response modulation and its possible preventive impact on autoimmune disorders [19,20]. In addition, prolactin has been linked to autoimmune disorder aggravation, which may account for the increased disease activity in females [21]. The notable variations in these hormone levels across genders highlight the possible impact of hormones on the severity and course of SLE illness.

Males had a slightly higher mean level (4.507) than females (4.076) in the analysis of anti-ds-DNA antibody levels, as shown in our research, but this difference was not statistically significant. This is in line with another study that reported gender-specific differences in anti-ds-DNA antibody levels; however, such differences are not always statistically significant [22]. Although blood prolactin and testosterone levels differ across genders, there seems to be a limited link between anti-ds-DNA antibody levels and prolactin and testosterone levels. This shows that the effect of hormones on anti-ds-DNA antibody levels may be complicated and dependent on other variables.

All things considered, these results advance our knowledge of the potential differential effects of sex hormones on SLE pathogenesis in men and females, emphasizing the need for more studies to clarify the underlying processes and corroborate these findings in a variety of demographic contexts.

Study limitations

When analyzing our study's findings, it is important to consider its limitations. First, the sample size of 90 patients may limit the generalizability of the conclusions, particularly due to the higher proportion of female participants, which could skew the results. Second, the cross-sectional design of the study limits the ability to establish causal relationships between hormone levels and anti-ds-DNA antibody levels. A notable limitation is the absence of a control group of healthy individuals, which would have provided a baseline for comparing hormone levels and further clarified their role in SLE pathogenesis. Additionally, the study focused on a specific geographic population, i.e., "Sudanese people", so the findings may not be applicable to other groups with different genetic, environmental, and hormonal backgrounds. The study also did not account for potential confounding factors such as medication usage and coexisting diseases that might influence hormone levels and disease activity. Finally, variations in the accuracy and sensitivity of the kits used to measure hormones and antibodies could impact the reliability of the results. Addressing these limitations in future research, including the inclusion of healthy controls, could offer a more comprehensive understanding of hormonal influences on SLE.

## Conclusions

Gender-specific variations in sex hormone levels and their correlation with anti-ds-DNA antibodies in SLE patients are explored in this research. Males have much greater amounts of prolactin and testosterone than females, which may indicate a possible hormonal effect on illness regulation. The poor associations between these hormones and anti-ds-DNA antibody levels, however, suggest that illness variability is probably caused by other causes. These results highlight the need for further investigation into the intricate relationships between sex hormones and the processes underlying autoimmune diseases across a variety of demographics.
